# Qualification of Standard Membrane-Feeding Assay with *Plasmodium falciparum* Malaria and Potential Improvements for Future Assays

**DOI:** 10.1371/journal.pone.0057909

**Published:** 2013-03-06

**Authors:** Kazutoyo Miura, Bingbing Deng, Gregory Tullo, Ababacar Diouf, Samuel E. Moretz, Emily Locke, Merribeth Morin, Michael P. Fay, Carole A. Long

**Affiliations:** 1 Laboratory of Malaria and Vector Research, National Institute of Allergy and Infectious Disease, National Institutes of Health, Rockville, Maryland, United States of America; 2 PATH Malaria Vaccine Initiative, Washington, District of Columbia, United States of America; 3 Biostatistics Research Branch, National Institute of Allergy and Infectious Disease, National Institutes of Health, Bethesda, Maryland, United States of America; The George Washington University Medical Center, United States of America

## Abstract

Vaccines that interrupt malaria transmission are of increasing interest and a robust functional assay to measure this activity would promote their development by providing a biologically relevant means of evaluating potential vaccine candidates. Therefore, we aimed to qualify the standard membrane-feeding assay (SMFA). The assay measures the transmission-blocking activity of antibodies by feeding cultured *P. falciparum* gametocytes to *Anopheles* mosquitoes in the presence of the test antibodies and measuring subsequent mosquito infection. The International Conference on Harmonisation (ICH) Harmonised Tripartite Guideline Q2(R1) details characteristics considered in assay validation. Of these characteristics, we decided to qualify the SMFA for Precision, Linearity, Range and Specificity. The transmission-blocking 4B7 monoclonal antibody was tested over 6 feeding experiments at several concentrations to determine four suitable concentrations that were tested in triplicate in the qualification experiments (3 additional feeds) to evaluate Precision, Linearity and Range. For Specificity, 4B7 was tested in the presence of normal mouse IgG. We determined intra- and inter-assay variability of % inhibition of mean oocyst intensity at each concentration of 4B7 (lower concentrations showed higher variability). We also showed that % inhibition was dependent on 4B7 concentration and the activity is specific to 4B7. Since obtaining empirical data is time-consuming, we generated a model using data from all 9 feeds and simulated the effects of different parameters on final readouts to improve the assay procedure and analytical methods for future studies. For example, we estimated the effect of number of mosquitoes dissected on variability of % inhibition, and simulated the relationship between % inhibition in oocyst intensity and % inhibition of prevalence of infected mosquitos at different mean oocysts in the control. SMFA is one of the few biological assays used in preclinical and early clinical development of transmission-blocking vaccines, and this study strongly supports its further development and application.

## Introduction

Continuous efforts to reduce malaria burden, such as application of insecticide treated nets, anti-malarial drugs and indoor insecticide spraying, have contributed to a decrease in mortality due to malaria, particularly due to *Plasmodium falciparum*, from an estimated 1.8 million deaths in 2005 to 1.2 million in 2010 [1]. However, to achieve the ultimate goal of malaria eradication, more effective tools will be required in view of the increasing resistance of malaria parasites and mosquito vectors to existing drugs and insecticides, respectively. Although vaccination is considered to be one of the most cost-effective control methods for a range of infectious diseases, to date only one malaria vaccine candidate against the pre-erythrocytic stages, the RTS, S vaccine, has shown encouraging clinical protection and a large phase 3 trial is underway in Africa [2]. There is increasing interest in a transmission-blocking vaccine (TBV) which is designed to induce antibodies in human hosts against sexual stage malaria antigens or to antigens found in the mosquito vector. The TBV-induced antibodies are ingested by *Anopheline* mosquitoes along with parasites in the blood and subsequently inhibit parasite development in the mosquito host. Several phase 1 trials have been done with TBVs, such as *Plasmodium falciparum* surface protein 25 (Pfs25) [3]. These existing TBV candidates are not optimal; either by inducing insufficient levels of functional antibodies in humans and/or by showing some safety concerns (the specific antigen/adjuvant combination [not the antigen *per se*] was thought to cause the adverse reactions) [3]. Since an ideal TBV should induce long-lasting and high levels of functional antibodies in all populations who transmit malaria, further development of effective and safe TBVs is required.

The standard membrane-feeding assay (SMFA) has been utilized widely to assess the transmission-blocking potential of test antibodies both in preclinical and clinical vaccine development (transmission-blocking refers to reduction in oocyst intensity throughout this manuscript unless specified, while further studies are required to determine the relationship to the prevalence in mosquitoes). In this assay, a mixture of cultured *P. falciparum* gametocytes and test antibodies (either serum or purified immunoglobulin) is fed to *Anopheles* mosquitoes through a membrane feeding apparatus, and approximately one week later the mosquitoes are dissected to enumerate oocysts in the midguts. As the assay is currently performed, there often is a poor concordance of data when the same samples are tested in independent assays, thus making interpretation difficult [4], [5].

A robust assay to measure biological activity is essential for vaccine development [6], [7]. If the SMFA can provide reliable and biologically relevant data, it can be used for preclinical and early clinical Go/No-go decisions. According to the International Conference on Harmonisation (ICH) Harmonised Tripartite Guideline Q2(R1) [8], up to seven characteristics need to be considered for assay validation depending on the type of assay: Specificity, Linearity, Range, Accuracy, Precision (Repeatability, Intermediate Precision and Reproducibility), Detection Limit, and Quantitation Limit (Table S1). The guideline is clear on the definitions of these terms as used for assay validation, though often the words are used less strictly in publications when assays are described. Therefore, to avoid confusion, we will use capitalized words throughout this manuscript when we use the words according the ICH guidelines. Unlike “assay validation”, there is no clear definition or guideline of “assay qualification”. Therefore, we use the word “qualification” to mean a partial validation; i.e., evaluate several, not all, characteristics of the assay. In the case of fluorescence-based measurements of parasitemia, a few studies have been done to evaluate several of the above characteristics [9]–[11]. On the other hand, for the more complicated SMFA, only a very limited number of studies have discussed Intermediate Precision (inter-feed variability), one of the important aspects of measurements of Precision in the SMFA [4], [12]. Churcher et al [13] have recently carried out an extensive study of SMFA, and this paper enhances and corroborates many of the analyses in that paper.

Of the seven characteristics listed in the Q2(R1) guidelines for assay validation, we decided to qualify the SMFA with respect to four characteristics. The first one is Precision, focusing specifically on Repeatability and Intermediate Precision. In the case of SMFA, Repeatability was determined by evaluating intra-feed variability, and Intermediate Precision by inter-feed variability. The second characteristic is Linearity: in the context of SMFA, this was determined by evaluating whether (a transformation of) the % inhibition result is directly proportional to (a transformation of) the concentration of transmission-blocking antibody. We also evaluated Range of the SMFA: i.e., the interval between the upper and lower levels of transmission-blocking activity in which the analytical procedure has a suitable level of Precision and Linearity. The fourth characteristic is Specificity: i.e., whether we can detect transmission-blocking activity of test antibody in the presence of unrelated antibodies which may be expected to be present in a test sample.

The ultimate goal is to establish a robust SMFA which can provide a biologically relevant means for making an informed Go/No-go decision (especially SMFA with human antibodies before performing large Phase 2 or 3 studies). While basic methodologies are similar, there are several differences in different laboratories (e.g., different haematocrits, different mosquitoes, etc.), and the impact of such differences on the final readout (i.e., % inhibition) is not clear. As an initial attempt, in this study we decided to evaluate the four characteristics listed above when the assay was performed by our current method. More specifically we focused only on the feeding portion of the assay. We generated a quantity of mouse 4B7 monoclonal antibody (mAb) to perform the qualification, since 4B7 mAb is directed to the Pfs25 antigen and has been well-characterized for its transmission-blocking activity [14]. In the qualification test, various concentrations of 4B7 mAb were tested in 3 independent experiments to evaluate the Precision, Linearity and Range of the assay. For the Specificity test, IgG from normal mouse sera was prepared and tested by SMFA with and without 4B7 mAb to assess whether the transmission-blocking activity was modified by the presence of normal mouse IgG. In addition to qualification of the assay as currently performed, we used the data from 9 feeding experiments (pre-qualification and qualification feeds) to generate a model of the SMFA and use it to estimate the impact of modifications to the assay design and analytical methods on the performance of the assay.

## Results

### Precision, linearity and range

**Figure 1:**
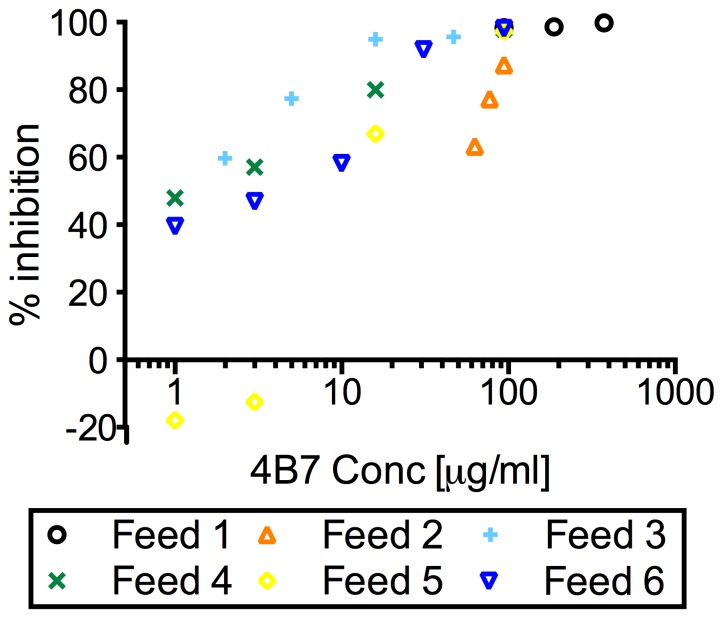
Dose dependent % inhibition in mean oocyst intensity by 4B7 mAb. Various concentrations of 4B7 mAbs (ranging from 1 to 375 µg/ml) were tested over 6 independent feeding experiments (Feed 1–6). Different symbols represent data from different feeding experiments.

In the pre-qualification experiments, serial dilutions of 4B7 mAb (ranging from 1 to 375 µg/ml) were tested over 6 independent feeding experiments (Feed 1–6) to determine suitable concentrations to use in the qualification experiments. In this series of experiments, each concentration of 4B7 mAb was tested in a single COM (container of mosquitoes, viz., a group of mosquitoes which were housed in the same container and were fed the same final mixture) in each feed. As shown in a previous study with 4B7 mAb [14], % inhibition of mean oocyst intensity (PIm) was dependent on 4B7 mAb concentration (Figure 1). Based on the results, we determined the four concentrations (1, 6, 23 and 94 µg/ml) of 4B7 mAb for the qualification experiments. These concentrations were expected to cover a range from 20 to 100% inhibition. Each concentration of 4B7 mAb and negative control were tested in triplicate (total of 15 COM) in each feed, and three independent feeds (Feed 7–9) were performed. The means (and standard deviations) of oocysts per mosquito in the control COM in the qualification experiments were 25 (10), 20 (10) and 23 (12) in Feed 7; 10 (7), 8 (5) and 10 (9) in Feed 8; and 23 (12), 26 (22) and 19 (25) in Feed 9. There are significant feed effects (p<0.0001) on the means, therefore, it is important to use the control from the same feed when calculating PIm. The number of mosquitoes without any oocysts were 1, 0 and 0 in Feed 7; 0, 0 and 1 in Feed 8; and 0, 0 and 5 in Feed 9.

**Figure 2:**
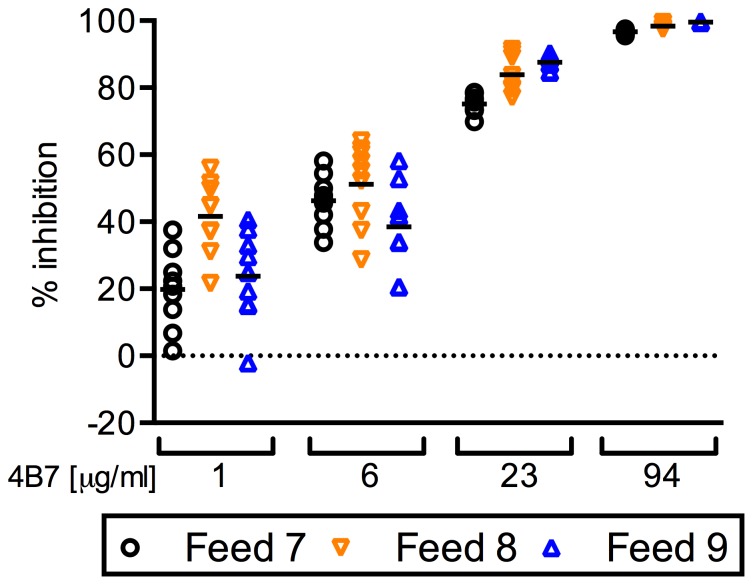
Intra- and inter-feed variability in PIm of 4B7 mAb. Four concentrations (1, 6, 23 and 94 µg/ml) of 4B7 mAb were tested in triplicate in each feed, and three independent feeds were performed (Feed 7, 8 and 9). Since there were 3 COM of negative control and 3 COM of 4B7 mAb at each concentration, 9 different numbers of PIm were calculated (individual dots) at each concentration in each feed. Bar represents the mean of the 9 calculated data.

We first evaluated two aspects of Precision: Repeatability (intra-feed variation) and Intermediate Precision (inter-feed variation). Since there were three COM for the negative control and three COM of 4B7 mAb at each concentration, 9 different values of PIm can be calculated at each concentration in each feed (Figure 2). Using U-statistics, we calculated Repeatability and Intermediate Precision (Table 1). There was a clear dose-effect on variance: lower doses of 4B7 mAb gave higher levels of variance. When intra-feed variance was compared with inter-feed variance at each concentration of 4B7 mAb in test-control match-up data (i.e., within each feed, PIm of the first test COM was calculated with first control COM, second test COM with second control COM, etc.), there were no significant differences at 1 and 6 µg/ml (p = 0.142 and 0.546 by an ANOVA test, respectively). However, at 23 and 94 µg/ml, inter-feed variances were significantly larger than intra-feed variances (p = 0.035 and 0.004, respectively). Similar results were obtained (data not shown) when we used different PIm data calculated from different combinations of test and control COM within a feed (e.g., PIm of the first test COM was calculated with second control COM, % inhibition of the second test COM with third control COM).

**Table 1:** Repeatability (intra-feed variance) and Intermediate Precision (inter-feed variance) of SMFA.

4B7^a^	Intra-feed variance^b^				Inter-feed variance^c^			
	Overall	Feed 7	Feed 8	Feed 9	Overall	7 & 8	7 & 9	8 & 9
1	189.6	172.8	157.0	239.0	261.2	347.2	145.5	291.1
6	164.7	77.3	197.1	219.5	150.8	103.8	129.3	219.3
23	17.1	10.7	35.4	5.1	52.1	54.2	82.2	20.0
94	0.5	0.6	0.9	0.0	2.6	2.0	4.6	1.1

a Concentration of 4B7 mAb in a feeder [µg/ml].

b Intra-feed variance estimates the variability between three PIm values (each one using one test COM and one control COM) where the test samples have the same 4B7 concentration and both PIm are measured on the same feed. We use U-statistics to estimate the intra-feed variance for each of the 3 feeds, as well as to estimate the overall estimate that combines the 3 feeds.

c Inter-feed variance is similar to the intra-feed variance, except that the variability is between two PIm values from identically concentrated test samples where one value is measured on one feed and the other value is measured on a second feed. Again we use U-statistics. We give the pairwise estimates and an overall estimate of inter-feed variance.

**Figure 3:**
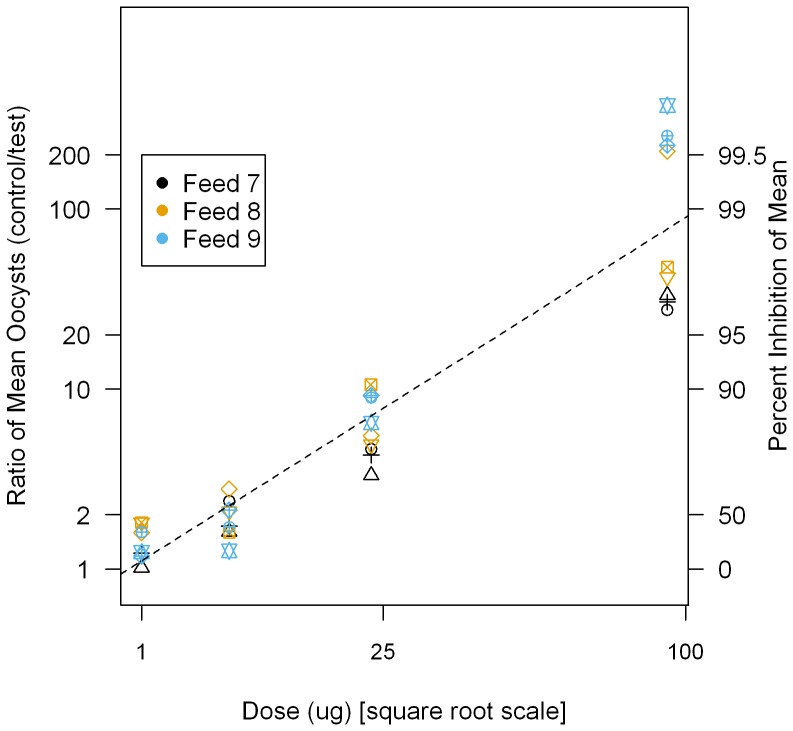
Relationship between 4B7 concentration and PIm. Various concentrations of 4B7 mAb were tested in the qualification experiments (Feed 7–9). For these data the first COM negative control is matched with the first COM of the 4B7 mAb at each concentration, the second with the second, etc. The square root of 4B7 concentration is shown on the x-axis, and the ratio of mean oocyst (mean of oocysts in control divided by mean of oocysts in test) is plotted on a log scale (shown on left side of y-axis, the associated PIm value is shown on the right side of the y-axis). Points with the same symbol use the same control, and points with the same color are from the same feed. Dotted line represents the best-fit line.

We then assessed Linearity of SMFA (whether a transformation of the test result is directly proportional to a transformation of the concentration of active antibody). When the square root of 4B7 concentration (x-axis) was plotted against the ratios of the mean between control and test on a log-scale (y-axis), the data were approximated by a linear relationship (Figure 3), supporting that the PIm measured in this assay was dependent on 4B7 concentration. We used the test-control match-up data and found that this linear model explained the relation well (R^2^ = 0.88, slope was significantly different from zero, p<0.0001).

While Linearity was demonstrated over the concentrations tested (Figure 3), the Precision varied over these same concentrations with the results indicating smaller inter-feed variations when 23 µg/ml and higher concentrations of 4B7 mAb were tested. These data suggest that at higher concentrations of 4B7 (i.e., 23 µg/ml and higher, which translates to >80% inhibition), it might be possible to obtain a reasonable estimate of PIm from a single feeding experiment. Therefore, we determined the Range (the levels of PIm with a suitable level of Precision and Linearity) of the SMFA to be when more than ∼80% inhibition results.

### Specificity

**Figure 4:**
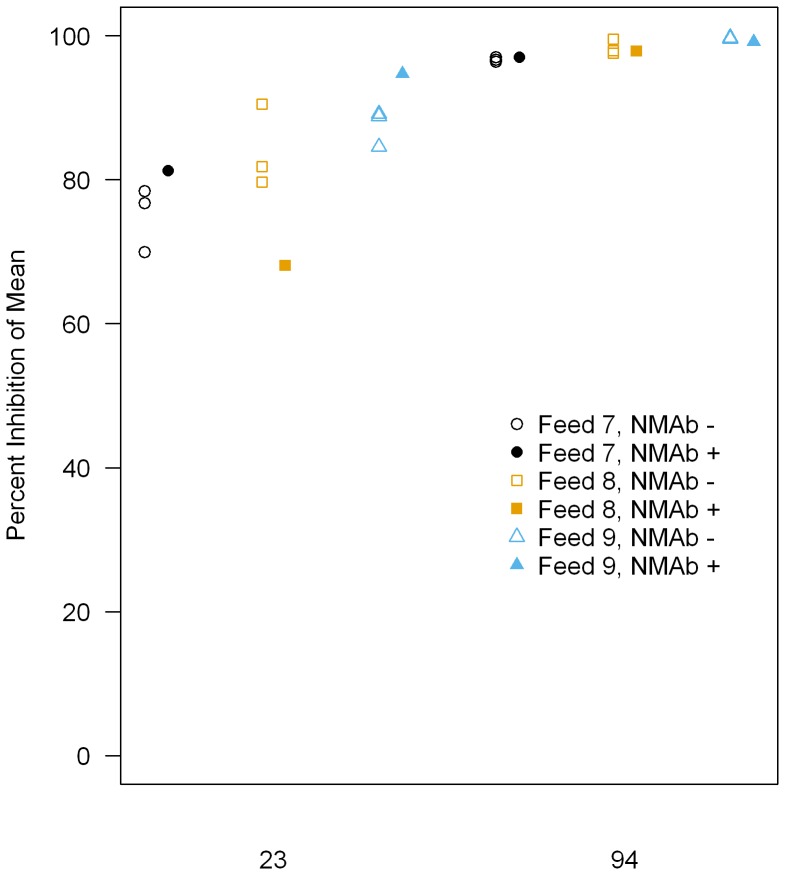
Effect of normal mouse IgG on 4B7 mAb. Two concentrations (23 and 94 µg/ml) of 4B7 mAb were tested with or without 0.75 mg/ml of normal mouse IgG (NMAb). PIm without NMAb were calculated 3 times for each feed (using 3 test COM and 3 separate control COM, 20 mosquitoes in each COM) and 1 time (1 test COM and 1 control COM, 20 mosquitoes each) for PIm with NMAb.

Specificity (whether we could detect transmission-blocking activity of test antibody in the presence of unrelated antibody which may be expected to be present in a test sample) was assessed to check whether this assay is useful to test mouse polyclonal antibodies in the future. We decided to test the 4B7 mAb at 23 and 94 µg/ml, with or without normal mouse IgG. The two concentrations of 4B7 were selected, since it is difficult to evaluate the effect of addition of normal mouse IgG if the 4B7 mAb itself shows variable PIm which would be the case at lower concentrations. As part of pre-qualification experiments, we tested normal mouse IgGs at concentrations ranging from 0.2 to 1.5 mg/ml. The normal mouse IgG showed 45% inhibition compared to the negative control (p = 0.019 by a Mann-Whitney test) at 1.5 mg/ml in a feed, while the same IgG showed insignificant inhibitions at the second highest dose tested, i.e., 0.75 mg/ml, in the two independent feeds (−8% inhibition, p = 0.946 in one feed; 10% inhibition, p = 0.217 in another feed). Based on those data, we decided to use a concentration of 0.75 mg/ml of normal mouse IgG to evaluate Specificity in the qualification experiments. When 23 µg/ml of 4B7 was tested in the presence and absence of normal mouse IgG, a mean PIm of the three feeds was 81.3% and 82.4%, respectively (Figure 4). At 94 µg/ml, a mean of 98.0% and 98.2% inhibition was observed, respectively. We used a linear model to statistically check the effect of the addition of normal mouse IgG at each concentration of 4B7 after controlling for feed effects. The normal mouse IgG changed the mean % inhibition by −0.8 (95% Confidence Interval, −11.4 to 9.7; p = 0.858) at 23 µg/ml, and −0.2 (95% CI, −1.1 to 0.7; p = 0.623) at 94 µg/ml. The data indicate that the addition of normal mouse IgG does not change the % inhibition of 4B7 mAb significantly, thus the activity of test antibody can be detected in the presence of 0.75 mg/ml of normal mouse IgG.

### Modelling

**Figure 5:**
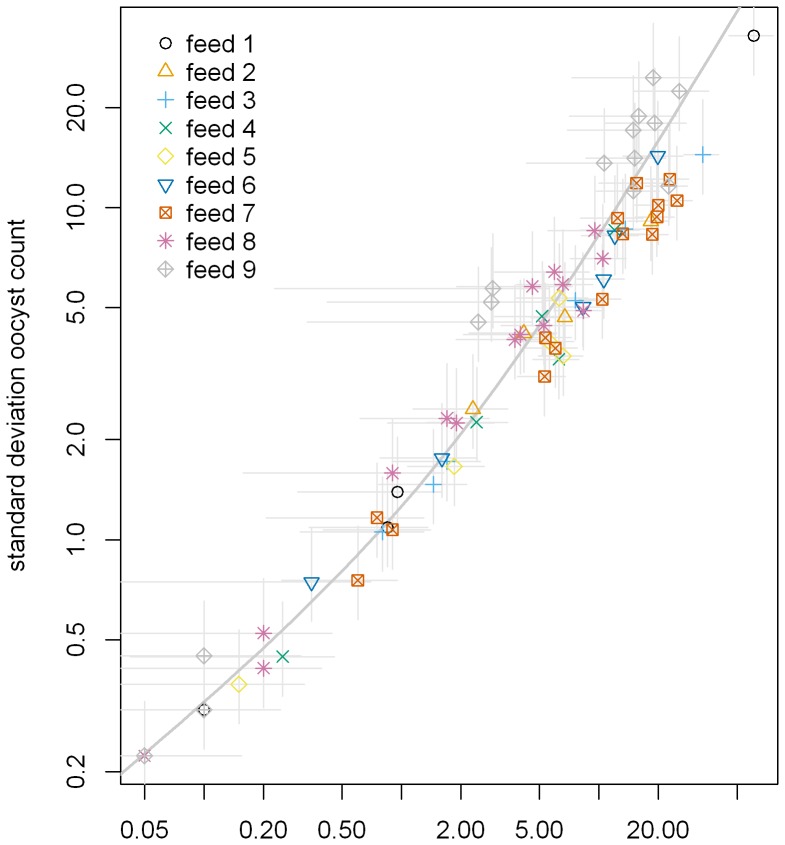
Relationship between mean number of oocysts and the standard deviation. For each COM, mean number of oocysts and standard deviation were calculated. Data from all COM tested in 9 independent feeding experiments are shown. Different symbols represent data from different feeding experiments and the line represents the best-fit curve from the zero-inflated negative binomial model. The R^2^ value for the fit is 0.94. Gray lines represent 95% confidence intervals calculated using the t-distribution (for the means) or chi square distribution (for the standard deviations).

We generated a model using the data from these 9 feeding experiments (pre-qualification and qualification feeds) to estimate the effect of modifications in the assay and/or analytical methods to guide future studies. As shown in Figure 5, there was a clear relationship between mean and standard deviation in all ranges of mean number of oocysts (including data both from negative control and 4B7 mAb tests), and mean-standard deviation relationship of the zero-inflated negative binomial model (see the line in Figure 5) fitted the data well. In addition, for the mean model we modelled the log mean oocyst value in a given feed as a function of feed effect and of the square root of antibody concentration (see Materials and Method section for details). The random effects for feed and COM, the zero inflation parameter, and the square root concentration effect were all highly significant (p<0.0001). The fit of this model can be examined by the data shown in Figure S1 where the data from all 9 feeds are plotted as Figure 3; the log of the ratio of mean oocyst count (y-axis) is the log of the mean oocyst counts for the control (which is a fixed value for each feeding experiment) minus the log mean oocyst count for the test.

**Figure 6:**
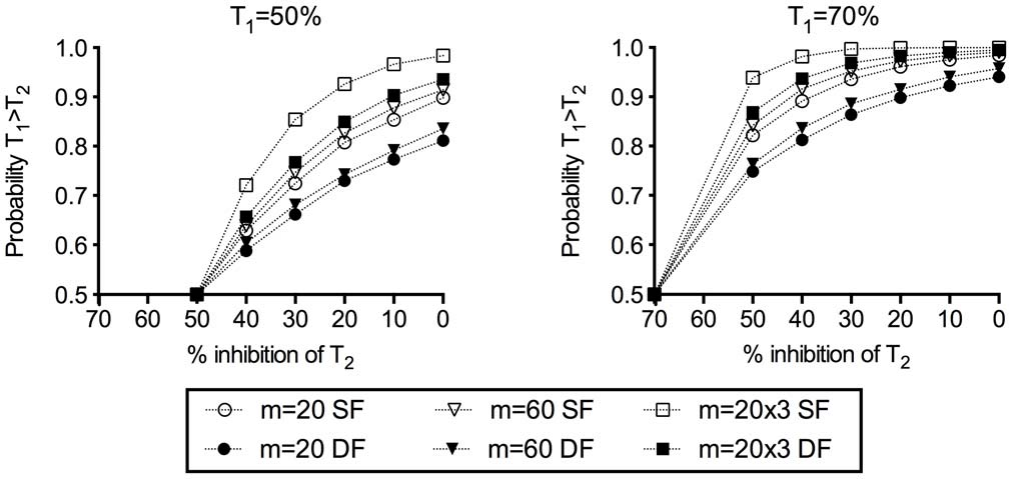
Effect of modifications of assay on the sensitivity of SMFA. In this simulation, we assumed there were two test samples (T_1_ and T_2_), and true PIm of T_1_ (50 or 70% inhibition compared to control) was higher than the true PIm of T_2_ (0, 10, 20, 30, 40 or 50%). Three different dissection conditions were simulated; 1) total of 20 mosquitoes were dissected from a single COM (m = 20), 2) total of 60 from a single COM (m = 60), and 3) total of 60, but from three COM (m = 20×3). In addition, we stimulated either: 1) T_1_ and T_2_ were tested in the same feeding experiment (SF), or 2) tested in different feeding experiments (DF). We assumed the mean number of oocysts in the control was 20. For each test condition, 10,000 data were generated to calculate the probability of feeds in which T_1_ showed higher PIm (i.e., lower mean oocyst number) than that T_2_.

We simulated how much variance could be reduced by dissecting more mosquitoes at each concentration of 4B7 mAb (Table 2). The modelling data indicated that dissection of 60 mosquitoes from a single COM rather than 20 mosquitoes would result in a significant reduction of variance at any concentration of 4B7 mAb. In addition, if a total of 60 mosquitoes were dissected from three different COM (20 mosquitoes per COM) rather than 60 mosquitoes from a single COM, additional reduction of variance was predicted from the model. We further assessed how such modification affected the sensitivity of the assay, where we evaluated sensitivity in this case by the proportion of times we could correctly determine which of two test samples had higher PIm. In this second simulation, we assumed there were two test samples (T_1_ and T_2_) and true PIm of T_1_ was higher than that of T_2_. In each given condition, the probability of feeds in which T_1_ showed higher PIm (i.e., lower mean oocyst number) than T_2_ was calculated using data from 10,000 simulations (Figure 6). We simulated multiple conditions. In terms of true PIm, T_1_ = 50 or 70% inhibition, and T_2_ = 0, 10, 20, 30, 40 or 50% inhibition were tested. For dissection, three different dissection scenarios were simulated: 1) total of 20 mosquitoes were dissected from a single COM (m = 20); 2) total of 60 from single COM (m = 60), and 3) total of 60, but from three COM (m = 20×3). In addition, we simulated either: 1) T_1_ and T_2_ were tested in the same feeding experiment (SF), or 2) tested in different feeding experiments (DF). For example, if the true PIm of T_1_ = 50% and T_2_ = 30%, 20 mosquitoes were modelled as from a single COM for each sample, and the two samples were tested in the same feeding experiment (m = 20 SF), the probability was calculated as 0.72 (Figure 6). As expected, the sensitivity of the assay was better when T_1_ and T_2_ were tested in the same feeding experiment (SF) than when tested in different feeding experiments (DF). While dissecting more mosquitoes from a single COM (m = 20 vs. m = 60) increased the probability of feeds in which T_1_ showed lower mean oocyst number than T_2_, the level of increase was less than 0.1. On the other hand, the difference in probability was larger when a condition where a total of 60 mosquitoes were dissected from 3 COM (m = 20×3) as compared to the other condition where 60 mosquitoes were dissected from a single COM (m = 60). We also evaluated the effect of the number of oocysts in the control in this simulation, but changing the mean of oocysts in the control from 4 to 30 had no noticeable effect on the probability (Figure S2).

**Table 2:** Estimated ratios of variances in SMFA.

4B7^a^	Condition 1		Condition 2		Expected Ratio of variance^d^
	Mosq^b^	COM^c^	Mosq^b^	COM^c^	
1	60	1	20	1	0.78
6	60	1	20	1	0.80
23	60	1	20	1	0.76
94	60	1	20	1	0.66
1	20	3	60	1	0.33
6	20	3	60	1	0.32
23	20	3	60	1	0.33
94	20	3	60	1	0.38

a Concentration of 4B7 mAb in a feeder [µg/ml].

b Number of mosquitoes dissected per Container of Mosquitoes (COM).

c Number of COM used.

d Average of variance (SMFA Condition 1)/variance (SMFA Condition 2) from 100,000 simulations. This many simulations ensures that we have 95% confidence that the estimates of the expected variance ratios are within 0.03 of their true values.

We then simulated 100,000 data sets from the model to estimate whether calculation of PIm using median of oocyst number was better than PIm represented as the arithmetic mean in terms of variance (Table 3). The model predicted that using arithmetic mean gave smaller variance at all four concentrations of 4B7 mAb tested, indicating that using arithmetic mean is better than median for calculation of PIm.

**Table 3:** Estimated variances of arithmetic mean and median methods^a^.

4B7^b^	Mean^c^	Median^d^ (n missing)	Expected ratio of variance^e^
1	2242	3039 (16)	0.74
6	746	1010 (6)	0.74
23	134	185 (7)	0.72
94	4.5	6.1 (7)	0.73

a Variances were calculated in the condition where 20 mosquitoes from a single COM were dissected.

b Concentration of 4B7 mAb in a feeder [µg/ml].

c Variance of PIm when calculated using arithmetic mean.

d Variance of PIm when calculated using median. The value “n missing” is the number out of 100,000 simulations with median = 0 in the control so that percent inhibition of a test could not be calculated.

e Average of variance (SMFA using mean)/variance (SMFA using median) from 100,000 simulations. This many simulations ensures that we have 95% confidence that the estimates of the expected variance ratios are within 0.04 of their true values.

**Figure 7:**
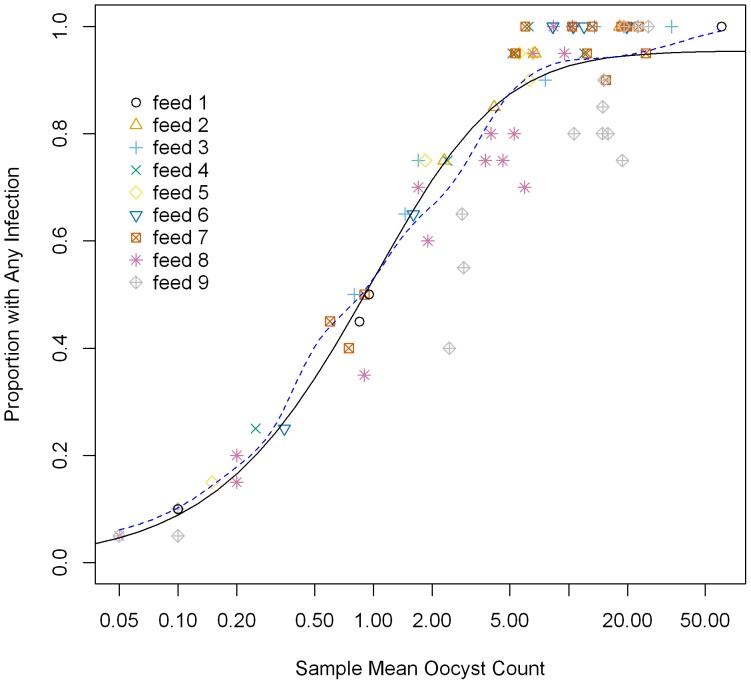
Sample mean of oocyst counts by proportion of mosquitoes with any infection. Each point represents one COM. Black line is the fit from the zero-inflated negative binomial model. The blue dotted line is a nonparametric moving window average (specifically, a kernel smoother with a normal kernel with bandwidth 0.5 log_10_ chosen to be slightly overfit).

**Figure 8:**
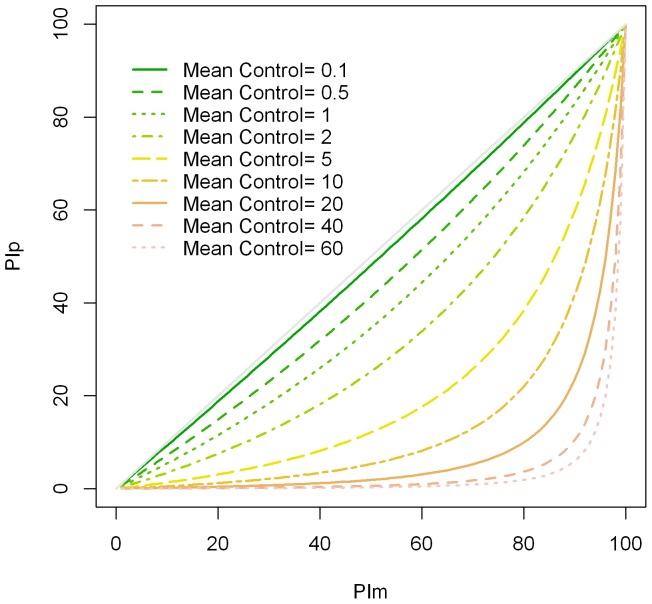
Effect of mean number of oocysts in the control on the two % inhibitions. The % inhibition of prevalence (PIp) is plotted against % inhibition in mean oocyst intensity (PIm) at different mean number of oocysts in the control.

We selected PIm as the main readout of SMFA in this study, however, % inhibition of prevalence (PIp, an increase in the proportion of mosquitoes that have no oocysts) also has been used in many other studies. Therefore, we assessed the relationship between PIm and PIp using the model. The adequacy of the negative binomial model was evaluated by plotting the mean oocyst count with the proportions of mosquitoes with any oocysts. We included the predicted proportion from the model together with a nonparametric smoother of the proportions (Figure 7). Medley et al [20] and others [13] have fitted the overdispersion parameter of the negative binomial model as a function of the mean, but we used a simpler approach. Although our zero-inflated negative binomial model used a simple constant for the overdispersion parameter, it appeared to adequately predict the proportions (Figure 7). We then modeled the relationship between the true values of PIm and PIp at different mean oocysts in the control under the zero-inflated negative binomial model (Figure 8). When the mean was equal to 0.1, the model specified that PIm and PIp values were similar. On the other hand, when the control had a mean of 60 oocysts, PIp was much lower than PIm for most values of PIm. The model indicates that PIp varies depending on the mean of oocysts in the control within samples with the same PIm. A similar result holds for the closely related model of Churcher et al [13].

## Discussion

In the present study, we qualified the SMFA using mouse 4B7 mAb and normal mouse IgG, as it is currently performed, in terms of its Precision (more specifically, Repeatability and Intermediate Precision), Linearity, Range, and Specificity. While the word “assay validation” has been used in quite a few publications for many assays, there are limited studies where each individual characteristic of a biological assay is assessed systematically. To the best of our knowledge, this is the first study to qualify multiple characteristics of the SMFA with respect to ICH Q2 (R1). In addition to the qualification of the assay, we generated a model to estimate the impact of modifications in the assay and to evaluate analytical methods for their utility in generating robust data.

According to the ICH Harmonised Tripartite Guideline Q2(R1), up to seven characteristics need to be considered for assay validation depending on the type of assay. The SMFA is one of a few biological assays widely utilized to test functional activity of antibodies both in preclinical and clinical vaccine development. However, the limited number of studies published to date have addressed a single validation parameter, Intermediate Precision (inter-feed variation) [4], [12]. Our previous studies with polyclonal antibodies [21], [22] indicated that it is difficult to assess Detection Limit and Quantitation Limit in our SMFA, due to the larger inter-feed variation at lower levels of PIm; therefore, we did not evaluate these characteristics. Indeed, the current study also showed that the lower concentrations of 4B7 mAb had larger variance in PIm (Figure 2 and Table 1). In addition, since at this time there is no widely accepted gold standard or procedure for SMFA, we cannot evaluate Accuracy (agreement between a conventional true value and an observed value) and Reproducibility (inter-laboratory variation). Therefore, we decided to partially validate (i.e., qualify) the assay. While the assay qualification with human antibodies is ideal, such human antibodies with strong transmission-blocking activity are not available at this moment. In addition, it is common to start preclinical vaccine development in mice. Thus, we used mouse antibodies in this study.

We evaluated Repeatability (intra-feed variability) and Intermediate Precision (inter-feed variability) at four concentrations of 4B7 mAb, which cover a range of % inhibition (from ∼20 to ∼100% inhibition). We have shown that variances in PIm were dependent on the concentration of 4B7 mAb. The data indicate that interpretation of results is difficult if a test sample has weak inhibitory activity, due to relatively large error of the assay, while we could obtain higher % inhibition consistently in any given feed if the test sample has strong activity. For the Linearity, the ICH Q2 (R1) recommends testing a minimum of 5 concentrations. However, since we have a practical limitation on the number of COM that can be comfortably handled in a single feeding experiment, we decided to test 4 different concentrations in order to evaluate Precision and Specificity at the same time. As shown in Figure 3 (and also in Figure S1), PIm measured in this study was dependent on 4B7 concentration, and there is a linear relationship when the square root of 4B7 concentration is plotted against the ratios of the mean between control and test on a log-scale (Linearity). We tested more than 5 different concentrations in the pre-qualification feeds (Figure S1), and the data support the Linearity of this assay. In terms of Range, while Linearity was demonstrated in the range of 20 to ∼100% inhibition (Figure 3), the Precision varied depending on the levels of activity (higher PIm results in smaller inter-feed variation). While we determined the Range of the SMFA, as currently performed, to be when more than ∼80% inhibition results, it is somewhat arbitrary. We note that the inter-feed variations change monotonically throughout the 4 values we explored. Therefore, it is possible to redefine (expand) the Range by testing samples with transmission-blocking activities between 49.4% (the mean % inhibition of 6 µg/ml of 4B7 mAb) and 80% inhibition (the mean of % inhibition of 23 µg/ml of 4B7 mAb). In addition, the ICH Q2 (R1) defines the Range as an interval between the upper and lower concentrations in which the analytical procedure has a “suitable level” of Precision, Accuracy and Linearity. However, the “suitable level” needs to be defined depending on the assay and no specific guidance is in place for the SMFA. Therefore, the Range can be determined differently depending on the usage of the assay. For the Specificity test, while we selected the normal mouse IgG concentration of 0.75 mg/ml for this study, similar to the discussion above, a different concentration of the normal mouse IgG (between 0.75 and 1.5 mg/ml) may show insignificant effect on PIm of 4B7 mAb. Since assay qualification is a continuous process, further studies are anticipated to support pre-clinical and clinical studies: for example, to refine Range and Specificity of SMFA, to determine the effect of mosquito species on variance in PIm, and to evaluate these characteristics for polyclonal antibodies from humans and animals.

The SMFA is a complex assay and many factors are considered to be possible sources of variability in the SMFA, such as the batch of human serum used in the gametocyte culture, temperature control (especially for late stages of gametocytes), and perhaps the size of mosquitoes. In this study we focused only on the feeding portion of the assay, as it is very difficult to evaluate all factors in a single study. We standardized the gametocyte culture as much as possible (e.g., culture volume, haematocrit, starting parasitemia, maintenance of temperature during medium change and feeding experiments) before starting this qualification study. We strictly followed our standard operating procedure to minimize the variation in gametocyte culture, preparation of samples and feeding experiment. In addition, we measured wing size of mosquitoes from four different COM and found that the coefficients of variation (CV) of wing size were 2.3–4.1%, which was much smaller than CV for the oocyst numbers (58.6–106.6%, data not shown). Despite efforts to standardize the assay, as it is well accepted, the mean numbers of oocysts in the control can still be highly variable; the mean in the control groups ranged from 5.6 to 60.7 in the 9 feeding experiments in this study. The qualification undertaken here was performed to improve the understanding of the uncertainty when interpreting data from both a single and multiple feeding experiments.

The SMFA is a labor-intensive assay and it takes about one month from starting a culture to the final oocyst counts. Therefore, generation of empirical data to judge the effect of assay and/or analysis modifications on the final readout requires considerable investment of effort and time. Hence, we tried to answer these questions using a model. In addition to our current study (Figure 5), a previous study by Medley et al. using *P. berghei* [20], showed that there was a strong relationship between mean number of oocysts and variation (or standard deviation). In addition, the current study with monoclonal antibody (Figures 3 and S1) and many other studies with sera from both animals and humans have shown that there are correlations between antibody level and PIm [3], [21]–[25]. Therefore, we believe our assumptions (e.g., variation of oocyst number in a group is dependent on the mean number of oocysts; % inhibition is dependent on antibody concentration) for generating the model are acceptable. As more empirical data are generated, the precision of each parameter in the model will be improved.

Many SMFA studies have used 30 or fewer mosquitoes per sample [12], [26]–[29]; however, Medley et al. suggested that using less than 50–100 mosquitoes per feed provides unreliable estimates of transmission-blocking activity using data from SMFA with *P. berghei* parasites [20]. We therefore estimated the effect of number of mosquitoes dissected using the model. By our model, increasing the numbers of mosquitoes dissected from 20 to 60 can significantly reduce the variability (Table 2) as expected. However, if the same total number of mosquitoes are dissected, it is better to dissect smaller numbers of mosquitoes from multiple groups (i.e., 20 mosquitoes each from 3 COM) rather than larger numbers from one group (i.e., 60 mosquitoes from 1 COM). The same conclusion is expressed in a different way by further simulation data (Figure 6). The simulation data shown in Figure 6 also indicate that the effect of larger numbers of dissected mosquitoes on sensitivity varies depending on the inhibitory activity of a test sample. For example, if T_1_ has 70% inhibitory activity, and T_2_ has 0%, even 20 mosquitoes from single COM gave more than a 90% probability of seeing a feed in which T_1_ showed higher PIm than that T_2_. Dissection with more mosquitoes did not dramatically increase this probability. However, showing that a test has larger mean intensity than a control is often not enough for pre-clinical and clinical vaccine development. If we wish to have a high confidence that two test samples have different activities, we will likely need to dissect more than one COM of 20 mosquitoes for the test and control.

We also used the simulation model to determine whether calculating PIm using median number of oocysts is better than using arithmetic mean number of oocysts. There was a strong relationship between the mean and standard deviation of oocyst number in each COM and the relationship fitted the zero-inflated negative binomial model (Figure 5). Therefore, it is not surprising that when we simulated under the zero-inflated negative binomial model, the arithmetic mean performed better than the median, because the sample mean was both the maximum likelihood estimator as well as the method of moments estimator for the mean parameter from the negative binomial model. A previous study by van der Kolk et al. indicated that using the arithmetic mean to estimate PIm gave smaller variance than using the geometric mean [4]. Taken together, these results suggest that it is better to use arithmetic mean to calculate PIm if the negative binomial model (or its zero-inflated version) holds true in the assay.

In this study, we used % inhibition in mean oocyst intensity (PIm) as the main readout of SMFA. Another readout, % inhibition of prevalence (PIp), an increase in the proportion of mosquitoes that have no oocysts, has also been used in many studies. Therefore, we modeled the relationship between PIm and PIp. The PIp readout is thought to be the best predictor of vaccine efficacy under field conditions, as it has been suggested that a single oocyst can generate a large number of infectious sporozoites [30]. However, we have selected PIm as the readout for our SMFA. One of the major differences between SMFA and natural infection is the mean of oocysts per mosquito. In direct feed assays (DFA), where mosquitoes feed directly on a malaria patient's skin [31]–[33], or in a study where mosquitoes were caught in the field [34], most of the mosquitoes had less than 5–6 oocysts. On the other hand, in the SMFA we usually have greater numbers of oocysts on average in the control group. The second point is that the currently used methods do not allow tight control of the resulting average number of oocysts in a control group in any given feeding experiment. Our data showed that PIm was reasonably consistent if a test sample had >80% inhibition (i.e., >23 µg/ml of 4B7 mAb) in the feeding experiments where the mean number of oocyst in the control groups varied from 8 to 26. On the other hand, the zero-inflated negative binomial model suggests that the same sample (with the same level of PIm) could show different levels of PIp in the different feeds if the mean number of oocysts in the control changed from 10 to 20 (Figure 8). The same sample may show a higher level of PIp when the mean in the control is 5 or less (the mean number of oocysts seen in the field). Since the mean numbers of oocysts in the control vary among different feeds, when the same sample is tested in multiple feeding experiments, we believe PIm provides more robust values (i.e., are less sensitive to changes in the control intensity) than PIp in the current format of SMFA (if the sample has a strong PIm activity). Further study is required to determine whether PIm measured by SMFA is a better predictor of efficacy in the field than PIp measured by SMFA when an efficacious transmission-blocking vaccine is developed.

Because antibodies are thought to be the major effectors blocking transmission from human host to mosquito vector, SMFA is one of the few biological assays by which the potential efficacy of transmission-blocking vaccine candidates may be evaluated. The direct membrane-feeding assay (DMFA) is another assay which has been used to measure biological activity of test antibodies. The DMFA utilizes patient blood as a source of gametocytes instead of cultured parasites in the SMFA. Therefore, the SMFA is considered to be a relatively better controlled assay compared to the DMFA. It is still controversial whether the data obtained by SMFA correlates with the data generated in DMFA [35], [36]. A recent study by Bousema et al [37] has shown a strong correlation of mosquito infection rate between the DMFA and DFA. However, no study has reported testing the correlation between DFA and SMFA in vaccinated people. Studies need to be done to determine what, if any, correlation exists. We believe the present work will strongly support such future studies, as it is very difficult to evaluate the correlation without knowing the range of error of these assays. Better understanding of the assay will also serve as the foundation for assay improvements going forward. In addition, we developed a model and simulated the effect of assay modifications and analytical procedures. The simulation data should promote future vaccine development, especially when a researcher wants to detect small differences among test samples. The model may be also used to generate new hypotheses which can be evaluated empirically later. Further studies are anticipated to assess whether our assumptions (e.g., variation of oocyst number in a group is dependent on the arithmetic mean number of oocysts; % inhibition is dependent on antibody concentration) for generating the model are reasonable when the control group has less than 4 oocysts, similar to the field situation where the most of the mosquitoes have zero or only a few oocysts.

## Materials and Methods

### Test material preparation

4B7 hybridoma cells were obtained from the Malaria Research and Reference Reagent Resource Center (MR4) as part of the Biodefense and Emerging Infections Resources Repository, National Institute of Allergy and Infectious Disease, National Institutes of Health: *Mus musculus* (B cell); *Mus musculus* (myeloma) 4B7, MRA-315, deposited by LH Miller, A Saul. The mAb was expanded in culture and purified by HPLC, dialyzed against 1× Phosphate Buffered Saline (PBS), concentrated to 0.5 mg/ml (the protein concentration was determined by a NanoDrop ND-1000, Thermo Science, Wilmington, DE), aliquoted and kept at −80°C until used. Normal mouse immunoglobulin G (IgG) was purified from normal mouse serum (Sanquin, Amsterdam, Netherlands) using protein G columns (Pierce, Rockford, IL) according to the manufacturer's instructions. The eluted IgG was dialyzed against 1×PBS, concentrated to 8 mg/ml, aliquoted and kept at −80°C until used. For each feeding experiment, the vials of 4B7 mAb and/or normal mouse IgG were freshly thawed, diluted with 1×PBS and used within a day.

### Standard membrane-feeding assay (SMFA)

Gametocyte culture of *P. falciparum* NF54 strain (initially provided by Dr. Steve Hoffman, Sanaria, Rockville, MD) was initiated at 0.15–0.2% asexual parasitemia and 5% haematocrit in 10 ml complete medium (RPMI-1640 with 6 g/L of HEPES, 50 mg/L of hypoxanthine, 2.5 g/L of sodium bicarbonate and 10% human serum). Three identical gametocyte cultures were maintained in an atmosphere of 5% O_2_, 5% CO_2_ and 90% N_2_ for 16–18 days with daily medium change. There was no addition of fresh uninfected erythrocytes, except on day 2 when the culture was divided to two cultures and fresh uninfected erythrocytes were added to them (giving a total of four cultures). For each feeding experiment, two or three cultures were selected based on their stage V gametocytemia and exflagellation activities and pooled. The average (standard deviation) of stage V gametocytemia in Feed 1–9 was 2.1 (0.6) %. The culture was centrifuged at 2000 g for 10 minutes, the medium of the mature gametocyte culture was replaced with normal human serum, and normal red blood cells (RBCs) were added to make a gametocyte mixture with 0.15–0.2% stage V gametocytemia at 50% haematocrit (0.5–1.3×10^5^/µl of stage V gametocytemia in the final feeder). Sixty µl of a test sample (a defined concentration of 4B7 mAb with or without normal mouse IgG in 1×PBS) was mixed with 200 µl of the gametocyte mixture, and the final mixture was immediately fed to ∼50 of 3–6 days old female *Anopheles stephensi* (Nijmegen strain) mosquitoes through a membrane feeding apparatus (18 mm diameter; Chemglass Life Sciences, Vineland, NJ). Throughout the paper, “Container of Mosquitoes” (COM) refers to a group of mosquitoes which were housed in the same container and were fed the same final mixture. In this study, antibody concentration (either 4B7 mAb or normal mouse IgG) of test in a feeding apparatus was calculated using the volume of liquid phase (i.e., 160 µl), not including the volume of RBCs (i.e., 100 µl). Mosquitoes were kept for 8 days and dissected (n = 20 per COM) to enumerate the oocysts in the midgut. Only midguts from mosquitoes with any eggs at the time of dissection were analysed. We judged an assay as acceptable when the negative control group (mosquitoes were fed without 4B7 or normal mouse IgG) had a mean (mean refers to an arithmetic mean throughout this manuscript) of 4 or more oocysts per mosquito, regardless of the number of mosquitoes without oocysts in the group. The human serum and RBC used in this study were purchased from Interstate Blood Bank, Inc. (Memphis, TN). Two different batches of pooled sera (pools of 17 or 23 individual sera) were used in this study (one pool for Feed 1–8 and the other pool for Feed 9). Different batches of RBC were used for different feeding experiments.

### Pre-qualification experiments

Serial dilutions of 4B7 mAb (ranging from 1 to 375 µg/ml) were tested over 6 independent feeding experiments (Feed 1–6). For normal mouse IgG without 4B7 mAb, concentrations ranging from 0.2 to 1.5 mg/ml of IgG were tested over 3 independent feeding experiments.

### Qualification experiments

Based on the data from the pre-qualification feeds, the concentrations of 4B7 mAb and normal mouse IgG to be used for the qualification study were determined. In each feeding experiment, four concentrations of 4B7 mAb (1, 6, 23 and 94 µg/ml) were tested in triplicate (total of 12 COM), and normal mouse IgG (0.75 mg/ml) was tested with or without 4B7 mAb (either 23 or 94 µg/ml) as single samples (total of 3 COM). As a negative control, a feed without any antibody was also tested in triplicate (total of 3 COM). Therefore, we used a total of 18 COM for each feeding experiment, and three independent feeding experiments were performed in the qualification study (Feed 7–9).

### Statistical analysis

Percent inhibition of mean oocyst intensity (PIm) was calculated as: 100×{1−(mean number of oocysts in the test)/(mean number of oocysts in the control)}.

Testing for feed effects on the mean oocysts in control groups was done by analysis of deviance from a quasi-Poisson model. In the qualification experiments, there were 3 COM of test for each concentration of 4B7 mAb and 3 COM of control within a feed. Thus, there were 9 different ways to estimate PIm. Because all 9 PIm values were not independent, we used generalized U statistics to combine them and got an unbiased estimate of Repeatability (intra-assay variability) and Intermediate Precision (inter-assay variability) [15] at each concentration of 4B7 mAb. To test whether inter-assay variability was larger than intra-assay variability, we did an Analysis of Variance (ANOVA) test to assess for feed effects on PIm within each concentration, using only the 3 test-control match-up data (i.e., within each feed, PIm of the first test COM was calculated with first control COM, second test COM with second control COM, etc.). To test the Linearity, we used a linear model on the transformed data and concentration using a generalized estimating equation to account for the correlation caused by using the same control within feed, adjusted for the small number of clusters [16]. For the Specificity test, we used linear models to check the effect of normal mouse IgG and feed-to-feed variation on PIm.

We then generated a model to estimate the effect of modifications in this assay (e.g., enumeration of oocysts from more mosquitoes per COM, testing more COM per feeding experiment) and to compare different analytical methods (e.g., use median instead of arithmetic mean). In this modelling, data from both the pre-qualification and qualification feeds (total of 9 feeds) were used, except for the feeding data with normal mouse IgG. We used a generalized linear mixed model (GLMM), specifically, a zero-inflated negative binomial model with random effects for both feed and COM. Let Y*_ijk_* be the oocyst count of the *k*the mosquito in the *j*th COM of the *i* feeding experiment, then the mean oocyst number of the COM is modelled as (1−π) μ_ijk_, where π is the zero inflation parameter, and μ_ijk_ is the random mean effect from the negative binomial portion of the model. The random mean is modelled as log (μ*_ijk_*) = μ+γ (C*_ij_*?1/2)+α*_i_*+δ*_ij_*, where μ is the overall mean effect, γ is the effect on the square root of 4B7 concentration, C*_ij_* is the 4B7 concentration, α_i_ is the normally distributed random effect for the *i*th feed, and δ*_ij_* is the normally distributed random effect for the *j*th COM of the *i*th feeding experiment. The significance of the random effects, the zero inflation, and the concentration effect are determined using analysis of deviance. To ensure that the simulated ratios in Tables 2 and 3 are close to the true values, we calculated the maximum distance from the estimate to either of the 95% confidence limits using percentile bootstrap intervals calculated with 2000 replications [17].

While PIm was the main readout of SMFA in this study, to evaluate the relationship between PIm and % inhibition of prevalence (PIp), the zero-inflated negative binomial model was used with dispersion parameter, θ, and the given mean numbers of oocysts in the control. PIp was calculated as: 100×{1−(proportion of mosquitoes with any oocysts in the test)/(proportion of mosquitoes with any oocysts in the control)}, where the proportions come from the zero-inflated negative binomial model.

All statistical tests were performed by R (version 2.15.2) or Prism 5 (GraphPad Software Inc, La Jolla, CA), with the GLMM models fit using the glmmADMB R package [18], [19], and the GEE small sample adjustment using the default settings of the saws R package [16]. Probability values less than 0.05 were considered significant.

## Supporting Information

Table S1 **Characteristics need to be considered for assay validation.** ^a^Specific measurement in the context of SMFA.
(DOCX)

Figure S1 **Relationship between 4B7 concentration and PIm.** Various concentrations of 4B7 mAb were tested in 9 independent feeding experiments (Feed # 1–9). The square root of 4B7 concentration is shown on the x-axis, and the ratio of mean oocyst (mean of oocysts in control divided by mean of oocysts in test) is plotted on a log scale (shown on left side of y-axis, the associated PIm value is shown on the right side of the y-axis). Points with the same symbol use the same control, and points with the same color are from the same feed. Dotted line represents the best-fit line.
(PDF)

Figure S2 **Effect of oocyst number in the control on the sensitivity of SMFA.** In this simulation, we assumed there are two test samples (T_1_ and T_2_), and true PIm of T_1_ (50 or 70% inhibition compared to control) is higher than the true PIm of T_2_ (0, 10, 20, 30, 40 or 50%). Three different control conditions were simulated; 1) mean number of oocysts in the control is 4 (Co = 4), 2) mean of 10 (Co = 10), and 3) mean of 30 (Co = 30). In addition, we stimulated either: 1) T_1_ and T_2_ are tested in the same feeding experiment (SF), or 2) tested in different feeding experiments (DF). We assumed 20 mosquitoes are dissected from a single COM. For each test condition, 10,000 data were generated to calculate the probability of feeds in which T_1_ showed higher PIm (i.e., lower mean oocyst number) than that T_2_.
(PDF)
