# Supplementary material for: Qualification of Standard Membrane-Feeding Assay with Plasmodium falciparum Malaria and Potential Improvements for Future Assays
Source: PLoS One. 2013 Mar 6;8(3):e57909. doi: 10.1371/journal.pone.0057909 (PMC3590281; doi:10.1371/journal.pone.0057909)
Supplement: Table S1 — Characteristics need to be considered for assay validation. aSpecific measurement in the context of SMFA. (DOCX) [file pone.0057909.s001.docx]

| Characteristics | SMFA^a^ |
| --- | --- |
| Specificity | Whether we can detect transmission-blocking activity of test antibody in the presence of unrelated antibodies which may be expected to be present in a test sample |
| Linearity | Whether (a transformation of) the % inhibition result is directly proportional to (a transformation of) the concentration of transmission-blocking antibody |
| Range | The interval between the upper and lower levels of transmission-blocking activity in which the analytical procedure has a suitable level of Precision and Linearity |
| Accuracy | Agreement between a conventional true value and an observed value |
| Precision |  |
| Repeatability | Intra-feed variability |
| Intermediated Precision | Inter-feed variability |
| Reproducibility | Inter-laboratory variability |
| Detection Limit | The lowest % inhibition can be detected (but not necessarily quantitated) |
| Quantitation Limit | The lowest % inhibition can be quantitatively detected |
