# Supplementary figures and images for: Qualification of Standard Membrane-Feeding Assay with Plasmodium falciparum Malaria and Potential Improvements for Future Assays
Source: PLoS One. 2013 Mar 6;8(3):e57909. doi: 10.1371/journal.pone.0057909 (PMC3590281; doi:10.1371/journal.pone.0057909)

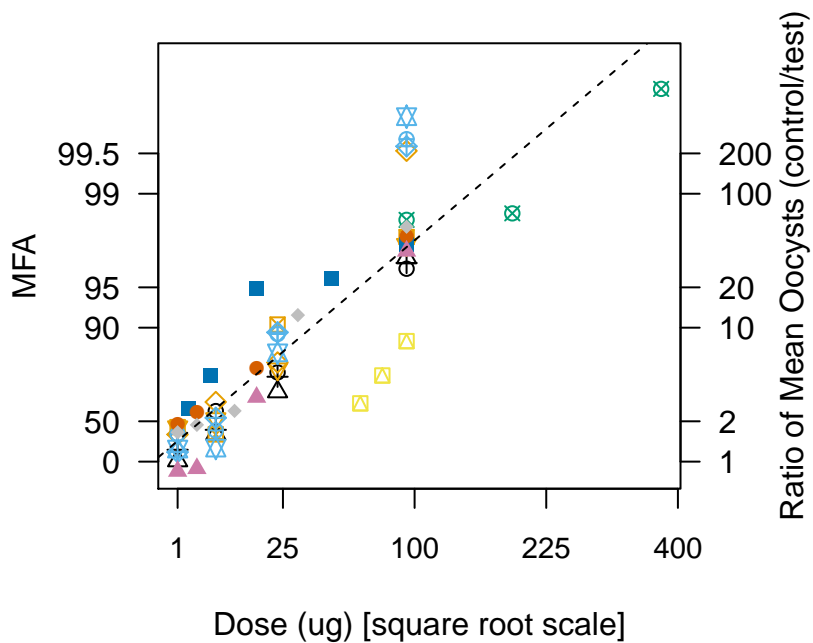

Supplement: Figure S1 — Relationship between 4B7 concentration and PIm. Various concentrations of 4B7 mAb were tested in 9 independent feeding experiments (Feed # 1–9). The square root of 4B7 concentration is shown on the x-axis, and the ratio of mean oocyst (mean of oocysts in control divided by mean of oocysts in test) is plotted on a log scale (shown on left side of y-axis, the associated PIm value is shown on the right side of the y-axis). Points with the same symbol use the same control, and points with the same color are from the same feed. Dotted line represents the best-fit line. (PDF) [file pone.0057909.s002.pdf]

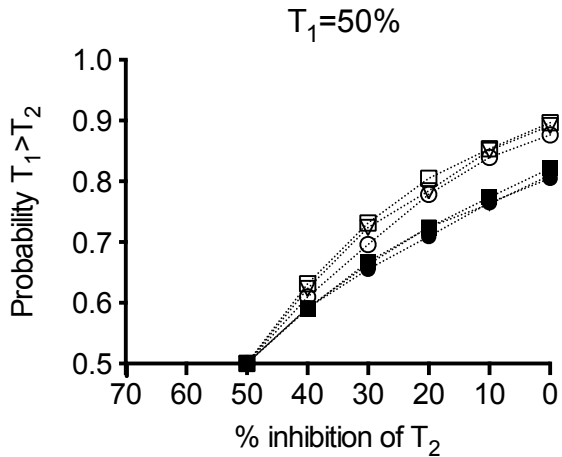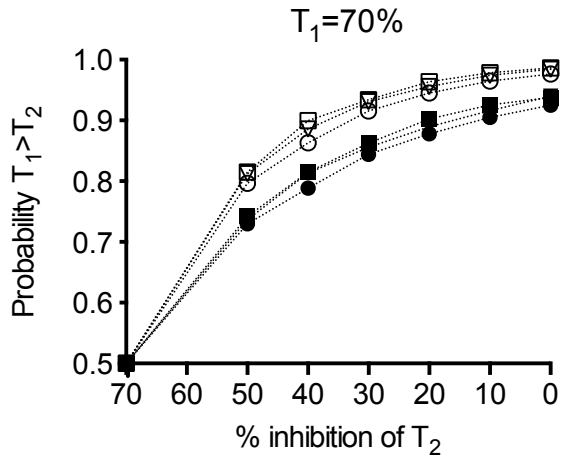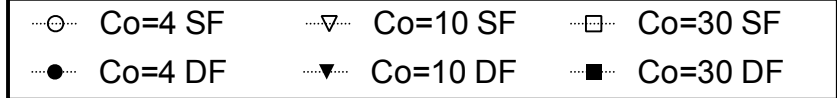

Supplement: Figure S2 — Effect of oocyst number in the control on the sensitivity of SMFA. In this simulation, we assumed there are two test samples (T1 and T2), and true PIm of T1 (50 or 70% inhibition compared to control) is higher than the true PIm of T2 (0, 10, 20, 30, 40 or 50%). Three different control conditions were simulated; 1) mean number of oocysts in the control is 4 (Co = 4), 2) mean of 10 (Co = 10), and 3) mean of 30 (Co = 30). In addition, we stimulated either: 1) T1 and T2 are tested in the same feeding experiment (SF), or 2) tested in different feeding experiments (DF). We assumed 20 mosquitoes are dissected from a single COM. For each test condition, 10,000 data were generated to calculate the probability of feeds in which T1 showed higher PIm (i.e., lower mean oocyst number) than that T2. (PDF) [file pone.0057909.s003.pdf]
